# Potential Role of Lisinopril in Reducing Atherosclerotic Risk: Evidence of an Antioxidant Effect in Human Cardiomyocytes Cell Line

**DOI:** 10.3389/fphar.2022.868365

**Published:** 2022-05-17

**Authors:** Lucia Scisciola, Rosaria Anna Fontanella, Surina, Giovanna Garofalo, Maria Rosaria Rizzo, Giuseppe Paolisso, Michelangela Barbieri

**Affiliations:** ^1^ Department of Advanced Medical and Surgical Sciences, University of Campania “Luigi Vanvitelli”, Naples, Italy; ^2^ Mediterranea Cardiocentro, Napoli, Italy

**Keywords:** ACE inhibitor, oxidative stress, human cardiomyocytes, SIRT-1, SIRT-6

## Abstract

The cellular mechanisms involved in myocardial ischemia/reperfusion injury (I/R) pathogenesis are complex but attributable to reactive oxygen species (ROS) production. ROS produced by coronary endothelial cells, blood cells (e.g., leukocytes and platelets), and cardiac myocytes have the potential to damage vascular cells directly and cardiac myocytes, initiating mechanisms that induce apoptosis, inflammation, necrosis, and fibrosis of myocardial cells. In addition to reducing blood pressure, lisinopril, a new non-sulfhydryl angiotensin-converting enzyme (ACE) inhibitor, increases the antioxidant defense in animals and humans. Recently, it has been shown that lisinopril can attenuate renal oxidative injury in the l-NAME-induced hypertensive rat and cause an impressive improvement in the antioxidant defense system of Wistar rats treated with doxorubicin. The potential effect of lisinopril on oxidative damage and fibrosis in human cardiomyocytes has not been previously investigated. Thus, the present study aims to investigate the effect of different doses of lisinopril on oxidative stress and fibrotic mediators in AC16 human cardiomyocytes, along with a 7-day presence in the culture medium. The results revealed that AC16 human cardiomyocytes exposed to lisinopril treatment significantly showed an upregulation of proteins involved in protecting against oxidative stress, such as catalase, SOD2, and thioredoxin, and a reduction of osteopontin and Galectin-3, critical proteins involved in cardiac fibrosis. Moreover, lisinopril treatment induced an increment in Sirtuin 1 and Sirtuin 6 protein expression. These findings demonstrated that, in AC16 human cardiomyocytes, lisinopril could protect against oxidative stress and fibrosis *via* the activation of Sirtuin 1 and Sirtuin 6 pathways.

## Introduction

Oxidative stress results from the unbalanced ratio between reactive oxygen production species (ROS) and inappropriate antioxidant capacity (Pizzino et al., 2017). Hyperglycemia is a critical factor associated with excessive, exaggerated ROS production (Akhigbe and Ajayi, 2021). In particular, it has been reported that hyperglycemia enhances intravascular ROS production through the activation of numerous enzymes such as those of mitochondrial respiratory chain, nicotinamide adenine dinucleotide phosphate (NADPH) oxidase, uncoupled endothelial nitric oxidase synthase (eNOS), and thioredoxin-interacting protein (TXNIP) (Granger and Kvietys, 2015) as such phenomena have been recognized to play a pivotal role in the genesis and development of atherosclerosis either at peripheral vascular or at cardiac levels (Hasheminasabgorji and Jha, 2021; Li et al., 2021).

Antioxidant agents have been the object of many investigations to find a neutralizing system that may reverse or counteract such an unbalanced ratio between pro-oxidative and anti-oxidative factors. The final target of such an effort is to prevent atherosclerotic-related diseases such as coronary heart diseases, heart failure with reduced ejection fraction, and peripheral artery disease.

Lisinopril is a non-sulfhydryl angiotensin-converting enzyme (ACE) inhibitor widely prescribed for treating patients suffering from hypertension and congestive heart failure, both tightly related to coronary heart disease. More recently, lisinopril has been shown to exert an antioxidant effect, lower lipid peroxidation, and anti-inflammatory effect in the kidney of Wistar rats previously treated with doxorubicin (Asaad et al., 2021). Whether lisinopril exerts its antioxidant effects in cardiac cells, which are more exposed to atherosclerosis and coronary heart diseases, is still a matter of debate (Oktem et al., 2011).

So far, in the present study, we investigate the effect of lisinopril as an antioxidant agent in AC16 culture cells of human cardiomyocytes and the relative impact as a positive epigenetic modulator of Sirtuin 6, thus hypothesizing that combined with the well-known anti-hypertensive effect, it may exert an anti-atherosclerotic role downgrading the intracellular oxidative stress.

## Materials and Methods

### Cell Culture

The AC16 human cardiomyocyte cell line was purchased from EMD Millipore (cat# SCC109). The cell line was tested and authenticated following the manufacturer’s instruction, and it was negative for mycoplasma contamination. Cells were cultured in Dulbecco’s modified Eagle’s medium (DMEM)/F12 (Microgem cat# AL215A) containing 10% fetal bovine serum (FBS, Euroclone cat# ECS0180L) and 1% antibiotics (penicillin-streptomycin) (Euroclone cat# ECB3001D) and 1% of L-glutamine (Euroclone cat# ECB3000D). Cells were incubated at 37°C with 5% CO_2_ over 7 days. Experiments were performed and repeated at least three times when the cell population reached 60%–70% confluence. Lisinopril (Sigma-Aldrich cat# L6292-100MG) concentration in culture was used 1 μM, 10 μM, and 100 μM and compared with 0 μM of lisinopril used as the normal control.

### Western Blot

Cells were dissolved in lysis buffer containing protease inhibitors (Tris HCl pH8 10 mM, NaCl 150 mM, NaF 10 mM, NP40 1%, PMSF 1 mM). Then, the proteins were subjected to 8% or 10% sodium dodecyl sulfate-polyacrylamide gel electrophoresis (SDS-PAGE) and transferred to 0.22 μm polyvinylidene fluoride (PVDF) membranes. The membranes were blocked with 5% non-fat milk in TBS-T (Tris-buffered pH8/0.15% Tween 20) at room temperature for 1 h, and then, incubated with primary antibodies diluted in with 5% non-fat milk in TBS-T (dilutions in according to datasheet), including antibodies against oxidative stress defense (Catalase, TRX) (Abcam, cat# ab179843), SOD2 (Abcam, cat# ab13533), NF-κB p65 (Abcam, cat# ab16502), TGF-β (Abcam, cat# ab 179695), osteopontin (Abcam, cat# ab8448), Galectin 3 (Abcam, cat# ab76466), BAX (Elabscience, cat# E-AB-22128), PDCD4 (Elabscience, cat# E-AB-52165), β-actin (Abcam, cat#ab8227), Vinculin (Elabscience, cat# E-AB-60433), and β-tubulin (Abcam, cat# ab6046) overnight at 4°C. After three washes in TBS-T, the membranes were incubated with corresponding secondary antibodies, horseradish peroxidase-conjugated anti-rabbit IgG, and horseradish peroxidase-conjugated anti-mouse IgG (GE Healthcare), for 1 h at room temperature. Immunocomplexes were visualized using Clarity Max Western ECL Substrate (Bio-Rad Laboratories, cat#1705062) and visualized using ChemiDoc Imaging System with Image Lab Software Version 6.1 software (Bio-Rad Laboratories). The molecular weight of proteins was estimated with prestained protein markers (ABM Opti-Protein-Marker cat# G623). Densitometry analysis was performed using ImageJ software.

## Results

**FIGURE 1:**
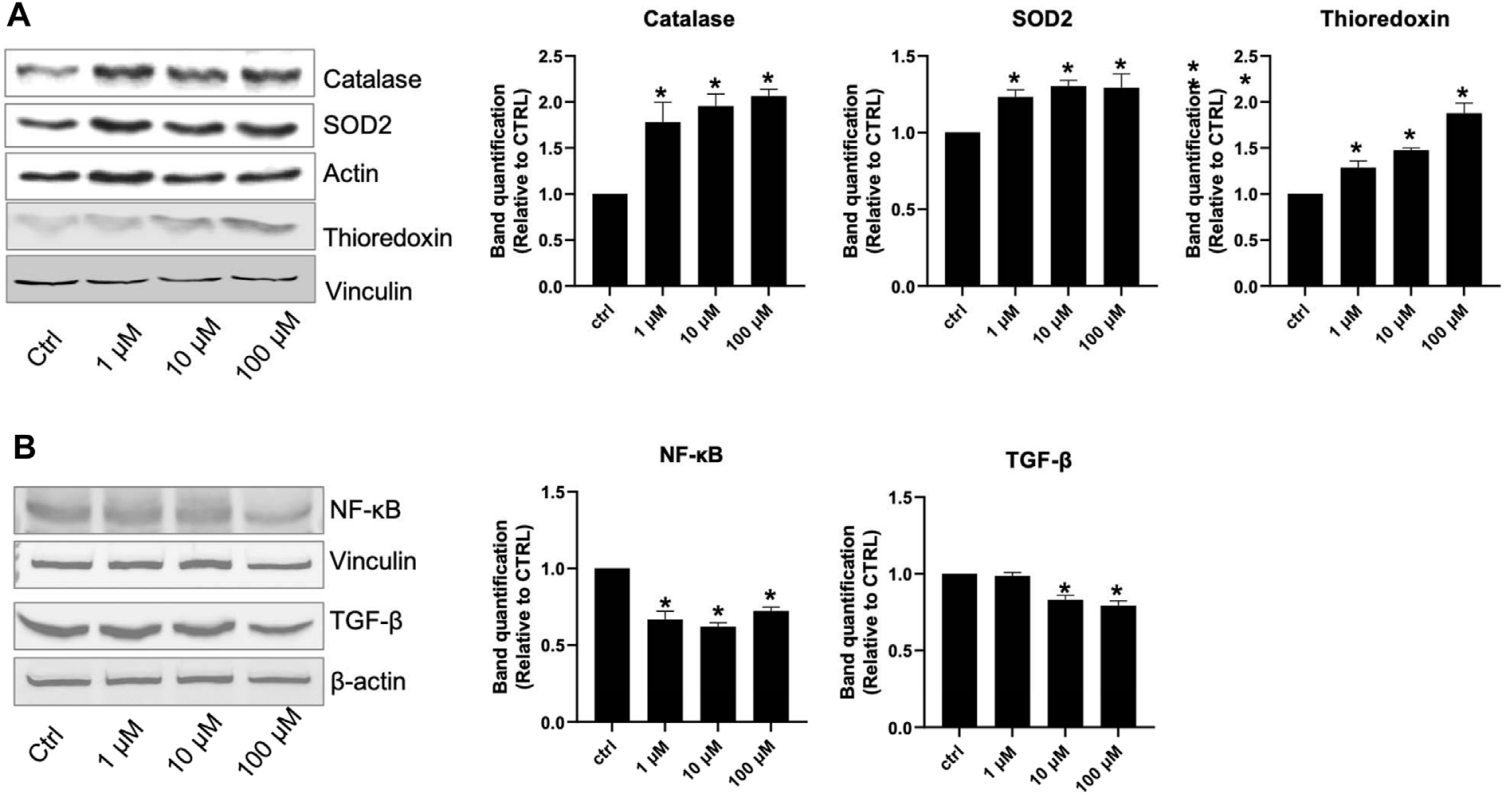
Effects of lisinopril treatment on proteins involved in oxidative stress defense and the inflammatory response. **(A,B)** Western blot analysis of proteins involved in oxidative stress response and inflammation. Data are mean ± standard errors. *p < 0.05 *vs.* CTRL.

**FIGURE 2:**
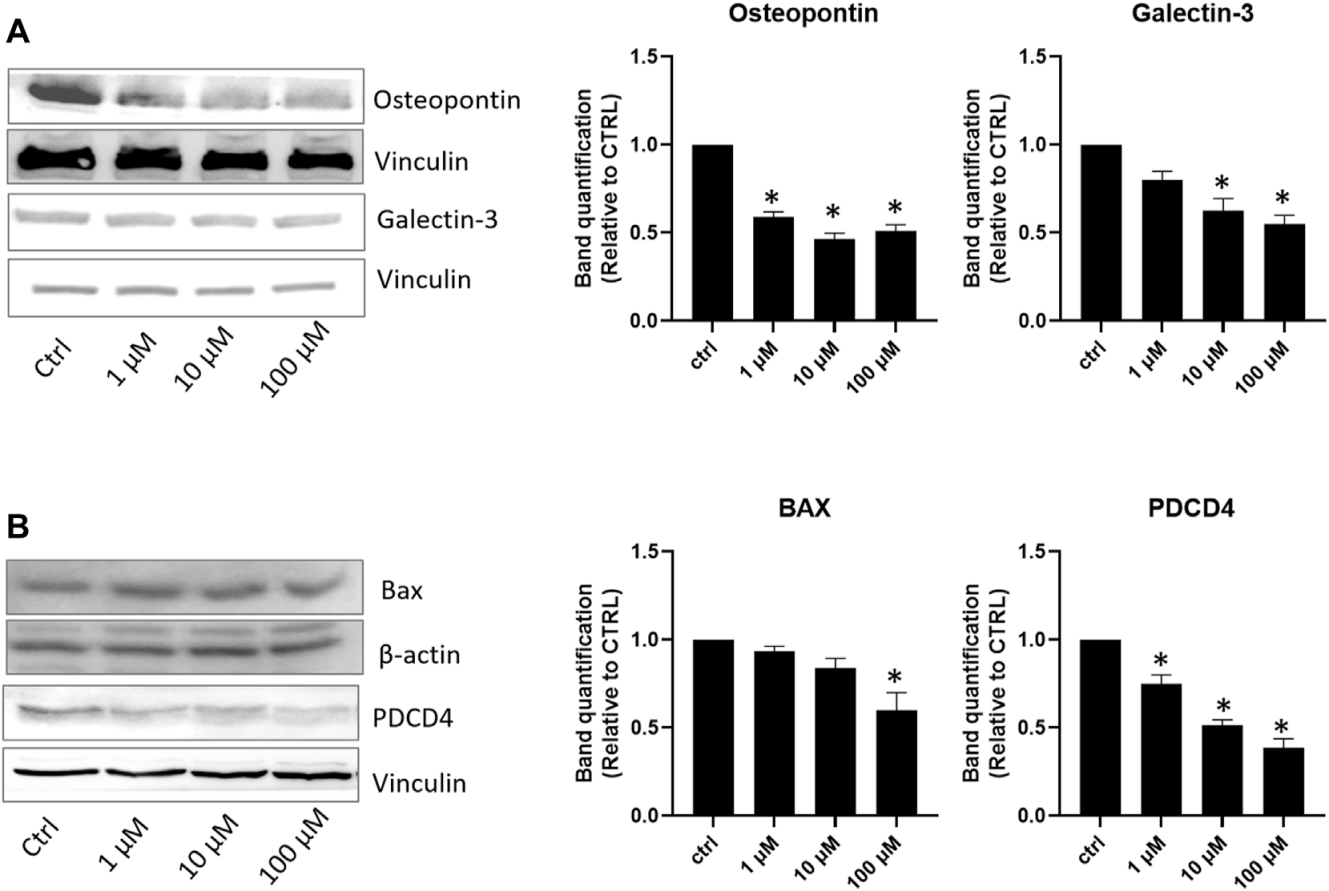
Effects of lisinopril treatment on proteins involved in fibrosis and apoptosis. **(A,B)** Western blot analysis of proteins involved in fibrosis and the apoptosis mechanism. Data are mean ± standard errors. *p < 0.05 vs. CTRL.

Seven days of incubation of AC16 cells on different lisinopril concentrations displayed a dose-curve effect. Despite the evidence that already 1 μM lisinopril was powerful enough to provide an antioxidant effect, incubation with 100 μM elicited the strongest response, as evidenced by the changes in catalase, SOD2, and thioredoxin levels (Figure 1A). The anti-inflammatory response tested by the changes in NF-kB and TGF-β paralleled the trend of antioxidant enzymes (Figure 1B). Noteworthy, the anti-atherosclerotic effect of lisinopril on AC16 human cardiomyocytes was completed by the evidence that such an ACE inhibitor produced a dose-dependent inhibition in osteopontin and Galectin-3 (Figure 2A) or in BAX and PDCD4 (Figure 2B) expression of anti-fibrotic and anti-apoptotic roles. For these latter two effects, a progressive inhibitory role of lisinopril was documented, with the highest dose (100 μM) having the strongest inhibition.

**FIGURE 3:**
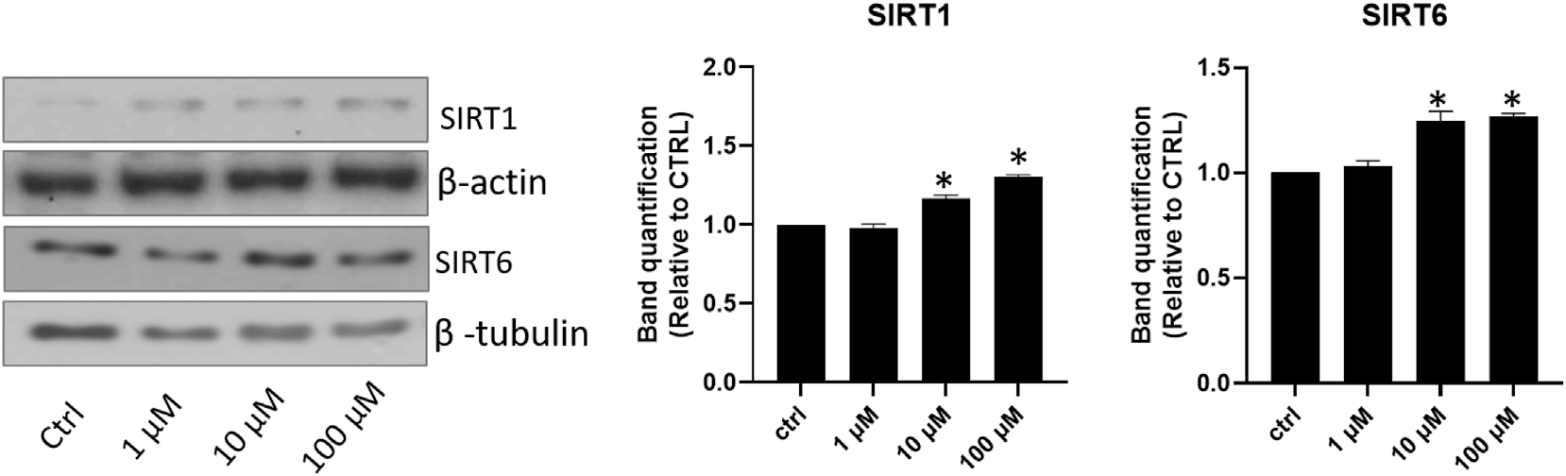
Effects of lisinopril treatment on Sirtuin 1 and Sirtuin 6. Western blot analysis of SIRT1 and SIRT6. Data are mean ± standard errors. *p < 0.05 vs. CTRL.

Due to the well-known anti-atherosclerotic effect of Sirtuins 1 and 6, dose-effect curves between lisinopril doses and Sirtuins 1 or 6 were investigated. Lisinopril addition to the incubation medium was associated with a significant increase in Sirtuin 1, which peaks at 10 μM and remains stable despite a further rise at 100 μM (Figure 3). In contrast, Sirtuin 6 displayed a precise dose-effect curve which had a progressive trend throughout the different doses investigated, with a maximum effect reached at 100 μM (Figure 3).

## Discussion

Lisinopril is an ACE inhibitor that has a potent peripheral vasodilator. Thus, it is primarily used for the well-known anti-hypertensive effect combined with anti-remodeling activity in patients affected by heart failure. In the present *in vitro* study, we provide evidence in AC16 human cardiomyocytes that lisinopril exerts anti-atherosclerotic effects as it is promoted in a dose-dependent manner: 1) an increase in antioxidant enzymes with a contemporary inhibition of anti-inflammatory, anti-apoptotic, and anti-fibrotic biomarkers and 2) a surge in Sirtuin 1 and Sirtuin 6 levels.

Several data have shown that free radical production has been associated with the development and progression of coronary atherosclerosis (Senoner and Dichtl, 2019). In particular, Li et al. (2021) demonstrated that circulating levels of total antioxidant status were associated with a more severe degree of coronary artery stenosis in elderly patients. Thus, one cannot exclude that drug administration with antioxidant power might help prevent atherosclerotic lesions’ development and progression. Lisinopril, a non-sulfhydryl ACE inhibitor, has been shown to exert scavenger action of free radicals and oxidants in male Wistar rats treated with doxorubicin to induce kidney injury (Asaad et al., 2021). In such experimental conditions, oral lisinopril administration was associated with a striking improvement in antioxidant defense that was impaired by simultaneous doxorubicin administration. It has been underlined that the lisinopril-mediated anti-oxidative effect might be related to the activation of prostacyclin synthesis (Li et al., 2021). In addition, lisinopril has been shown to upregulate constitutive e-NOS *via* an increase in bradykinin (Ancion et al., 2019) or downgrade lipid peroxidation (Silva et al., 2020). Cellular SOD and catalase content protect against the toxic effect of superoxide radicals; so far, lisinopril-associated stimulation in anti-oxidative cellular defense can be correlated with the anti-hypertensive outcome anti-atherosclerotic process (Sorriento et al., 2018).

Interestingly enough, lisinopril addition to the cellular medium was also associated with anti-apoptotic and anti-fibrotic effects; both phenomena strongly related to the anti-atherosclerotic role (Hasheminasabgorji and Jha, 2021). As far as the anti-fibrotic effect of lisinopril, previous experimental evidence demonstrated that free radical generation is associated with fibrosis due to activation in NADPH oxidase and production of growth factors (Oktem et al., 2011), both phenomena contrasted by lisinopril (Oktem et al., 2011; Saber et al., 2018; Asaad et al., 2021). Angiotensin II stimulates the NADPH oxidase to produce superoxide and hydrogen peroxide, but lisinopril inhibits the conversion of angiotensin I to angiotensin II, and thus the angiotensin II pro-oxidative effect was downgraded.

For the first time, our study demonstrated that lisinopril induced an increase in antioxidant enzymes with a contemporary inhibition of anti-inflammatory, anti-apoptotic, and anti-fibrotic biomarkers in human AC16 cardiomyocytes.

We acknowledge that the *in vitro* perspective of the evidence is a weakness of our study, but this is mitigated by several *in vivo* data that demonstrated the beneficial effects of lisinopril in contrasting oxidative stress and fibrosis at the cardiac level. In particular, in a study conducted on patients with acute coronary syndrome and concomitant diabetes mellitus type 2, lisinopril induced changes in antioxidant defense, increasing catalase activity, lowering blood creatinine, and eliminating protein in the urine (Kratnov et al., 2005a; Kratnov et al., 2005b). Moreover, in hypertensive African Americans with left ventricular hypertrophy, ACE inhibitors increase anti-fibrotic biomarkers (Romero et al., 2021).

More intriguing, our study demonstrated that lisinopril is associated in a dose-dependent manner with an intracellular increase in both Sirtuin 1 and Sirtuin 6.

Interestingly, both Sirtuins are best characterized for their protective roles against inflammation, vascular aging, heart disease, and atherosclerotic plaque development (D’Onofrio et al., 2018; Kuang et al., 2018). In particular, both Sirtuin 1 and Sirtuin 6 exert an atheroprotective effect by increasing NO production, blocking the NF-kB mediated inflammatory processes and consecutive pro-inflammatory cytokine expression, reducing oxidative stress, and preventing cellular senescence and apoptosis stabilizing telomeres (Merksamer et al., 2013; Kida and Goligorsky, 2016; Vachharajani et al., 2016; Toulassi et al., 2021). Moreover, Sirtuin 1 reduces plasma Pcsk9 levels, thereby increasing hepatic low-density lipoprotein-cholesterol receptor density and thus decreasing plasma low-density lipoprotein-cholesterol levels. Likewise, Sirtuin 6 represses triglyceride synthesis and fat metabolism, promotes fatty acid β-oxidation, and maintains low levels of low-density lipoprotein (LDL) cholesterol by deacetylating H3 at Lys-9 (H3K9) in the promoter of several genes involved in these metabolic processes (Akhigbe and Ajayi, 2021). These effects improve endothelial dysfunction and decrease atherosclerosis (Winnik et al., 2015).

Thus, although our *in vitro* experiment does not allow explaining the exact mechanisms by which lisinopril may exert the anti-atherosclerotic effects observed, results showing an increase in Sirtuin 1 and Sirtuin 6 levels following lisinopril treatment suggest that the beneficial effects induced by lisinopril may be related to an upregulation of Sirtuins expression. In agreement with previous data, in our study, the elevated levels of Sirtuins were associated with NF-kB Levels.

According to our results, in other systems, it is already known that SIRT1 is a direct target of the RAS pathway and that ACE inhibitors increase its expression. In particular, in osteoblast, ACEII induces mitochondrial oxidative stress and mtDNA damage by inhibition of the SIRT1–FoxO3a–MnSOD pathway (Li et al., 2014), and in aged rodents, the suppression of AngII/AT1/NADPH oxidase axis and RAS activation has been associated with SIRT1 upregulation (Diaz-Ruiz et al., 2015). Moreover, perindopril, a member of the angiotensin-converting enzyme inhibitor family, attenuates methotrexate-induced intestinal injury in rats, increasing SIRT1 expression (Sayed et al., 2022).

Among the hypothesized mechanisms to be explored with further studies, it was demonstrated that, in the HUVEC cell line, zofenoprilat, another member of the ACE inhibitors family, counteracts angiotensin II-mediated damage by restoring the SirT1 protein expression and its nuclear accumulation by inhibiting p38 which sequesters SIRT1 into the cytoplasm and negatively controls its expression (Marampon et al., 2013).

Further experiments are necessary to better clarify in human cardiomyocytes how lisinopril increases the expression of Sirtuins and if this mechanism might also include epigenetic changes.

In conclusion, lisinopril addition in the cellular medium for the growth of human cardiomyocytes is associated with a significant intracellular potentiation of antioxidant defense combined with an anti-fibrotic and anti-apoptotic effect. Because oxidative stress and fibrosis have been widely recognized to play a pivotal role as pro-atherosclerotic physio-pathological components, a lisinopril anti-atherosclerotic role combined with the anti-hypertensive action cannot be ruled out.

Whether the lisinopril-related anti-atherosclerotic action is a biologic effect with relevant clinical significance remains an object of further and focused randomized clinical trials.

## Data Availability

The raw data supporting the conclusion of this article will be made available by the authors without undue reservation.
